# Adsorption of dissolved aluminum on sapphire-c and kaolinite: implications for points of zero charge of clay minerals

**DOI:** 10.1186/1467-4866-15-9

**Published:** 2014-06-19

**Authors:** Johannes Lützenkirchen, Ahmed Abdelmonem, Rohan Weerasooriya, Frank Heberling, Volker Metz, Remi Marsac

**Affiliations:** 1 Karlsruhe Institute of Technology (KIT) Institut für Nukleare Entsorgung, INE Hermann-von-Helmholtz-Platz 1, 76344 Eggenstein-Leopoldshafen, Germany; 2 Karlsruhe Institute of Technology (KIT) Institute of Meteorology and Climate Research - Atmospheric Aerosol Research – (IMKAAF), Hermann-von-Helmholtz-Platz 1, 76344 Eggenstein-Leopoldshafen, Germany; 3 Department of Soil Science, University of Peradeniya, Peradeniya, Sri Lanka

**Keywords:** Isoelectric point, Streaming potential, Second harmonic generation, Electrophoretic mobility, Kaolinite, Sapphire, Alumina

## Abstract

We have studied the impact of dissolved aluminum on interfacial properties of two aluminum bearing minerals, corundum and kaolinite. The effect of intentionally adding dissolved aluminum on electrokinetic potential of basal plane surfaces of sapphire was studied by streaming potential measurements as a function of pH and was complemented by a second harmonic generation (SHG) study at pH 6. The electrokinetic data show a similar trend as the SHG data, suggesting that the SHG electric field correlates to zeta-potential. A comparable study was carried out on kaolinite particles. In this case electrophoretic mobility was measured as a function of pH. In both systems the addition of dissolved aluminum caused significant changes in the charging behavior. The isoelectric point consistently shifted to higher pH values, the extent of the shift depending on the amount of aluminum present or added. The experimental results imply that published isoelectric points of clay minerals may have been affected by this phenomenon. The presence of dissolved aluminum in experimental studies may be caused by particular pre-treatment methods (such as washing in acids and subsequent adsorption of dissolved aluminum) or even simply by starting a series of measurements from extreme pH (causing dissolution), and subsequently varying the pH in the very same batch. This results in interactions of dissolved aluminum with the target surface.

A possible interpretation of the experimental results could be that at low aluminum concentrations adatoms of aluminum (we will refer to adsorbed mineral constituents as adatoms) can form at the sapphire basal plane, which can be rather easily removed. Simultaneously, once the surface has been exposed to sufficiently high aluminum concentration, a visible change of the surface is seen by AFM which is attributed to a surface precipitate that cannot be removed under the conditions employed in the current study.

In conclusion, whenever pre-treatment or the starting point of an experiment favor the dissolution of aluminum, dissolved Al may remain in the experimental system and interact with the target surfaces. The systems are then no longer pristine and points of zero charge or sorption data are those of aluminum-bearing systems.

## Background

There is a huge discrepancy in reported points of zero charge (PZCs) for clay minerals based on the compilations by Kosmulski (the reader is referred to these references for more detailed information) [1,2]. This is partially due to the fact that PZCs determined from potentiometric titrations (points of zero net proton charge, PZNPCs) and isoelectric points (IEPs) from electrokinetic measurements are different properties. The different PZCs as obtained from different measurement principles are not necessarily related even conceptually. This becomes particularly clear in the case of clays and clay minerals. Thus, in the case of kaolinite/kaolin Kosmulski [1] reports literature PZNPCs that range from pH 2.2 to 7.5, while IEPs are below pH 5. In general for clays the PZCs from titrations (that include single potentiometric titrations, common intersection points and mass titration endpoints) are higher than the IEPs. The significant difference in the results from those two techniques may be explained as follows:

While the potentiometric titrations test the interactions of the surface with protons and hydroxide ions, electrokinetics is a measure of the net charge within the shear-plane and this includes all contributions. Potentiometric titrations miss the permanent negative charge on a number of minerals that appears to dominate the electrokinetic potential. Although the IEPs appear to be more consistent compared to the results from titrations, the reported range of IEPs remains surprisingly broad and relatively little has been done to evaluate possible reasons. Typical candidates to explain differences in IEP include sample origin [1,2], sample preparation [3], particle size [4], and experimental procedures [5]. However, for a prominent kaolinite sample (KGa-2, grain size < 1 μm) the IEPs in Kosmulski’s collection [1] vary from 2.9 to 4.8. A series of measurements on Chinese kaolinites with one instrument yields IEPs from pH 2.8 to 4.3. For these two examples the origin of the samples and instrumental issues can be disregarded as potential causes for differences. Size comes into play at too small dimensions (<10 nm) [4,6] to be relevant for the particles discussed here. The present work focuses on one potential aspect of sample preparation and experimental procedure, namely the fact that clays have a finite solubility that depends on pH. This finite solubility causes the release of silica and alumina as major solutes that are known to specifically adsorb to oxide surfaces [7,8]. Obviously, dissolution rates will determine the ultimate concentrations in solution. Dissolution may also be incongruent. Re-adsorption processes would counter-act the kinetically controlled dissolution process. In principle, adsorption of sorbent component ions to that very sorbent can also be seen from the point of view of solubility. The study of such systems is inherently difficult if both solubility and adsorption phenomena are involved, because it is not straightforward to distinguish contributions from dissolution/precipitation and adsorption/desorption. In a study on hematite, adsorption of ferric iron to the basal plane was reported [9], a rare case where such phenomena have been observed directly. The “adatoms” as these authors termed those particular “surface complexes” were released from the surface when lowering the pH. The extents of both equilibrium desorption and dissolution in this case will vary with pH. Extreme pH values are expected to cause enhanced dissolution. The use of the term adatoms by Eggleston et al. [9] implies that those authors favor an adsorption process to interpret their observations. However, ultimately if no phase transformation occurs and in case sufficient sorbent is available, the system will be controlled by solubility constraints.

Our interest is in the potential role of adatoms. By using single crystals of sapphire, we can probe the interaction with intentionally added aluminum in a controlled way. Due to the very low surface area to solution volume ratio, the aluminum concentration in the system corresponds to the added aluminum. Dissolution from the sample if it occurred to a significant extent would hardly affect aqueous aluminum concentration. Streaming potential experiments [10,11] conducted over the last decade for sapphire-c single crystals have shown that in the absence of added aluminium results were highly reproducible on one set of samples, whether experiments were repeated either in one solution or in a fresh solution. In the more complicated case of clays it is difficult to make a statement about the extent of additional interference by dissolved silica. The present study focuses solely on the effect of dissolved aluminum. Addition of aluminum and use of time scales that would avoid strong dissolution of our kaolinite restricts the observed effects to the action of the aluminum. A realistic scenario, with dissolution at low pH and subsequent increase of the pH would involve simultaneous interaction of the dissolved components. The interaction of aluminum ions with montmorillonite has been previously discussed by Bruggenwert et al. [12] who also provide a concise overview on the interactions of aluminum and clay minerals. While the main discussion is about the effect of the clays on aluminum, the issue of irreversibly bound aluminum is also discussed. As mentioned above Eggleston et al. [9] observed adatoms of Fe(III) on the basal plane of hematite. These adatoms could be rather easily removed in that study. The basal plane of hematite is structurally equivalent to that of sapphire, which in turn is related to kaolinite and gibbsite faces. The presence of adatoms might contribute to a number of features, including distinct differences in surface charging of gibbsites [13] or effects observed on hematite single crystal electrodes [14,15]. The charging effects will have important consequences. One effect is immediately related to the interaction of water with these surfaces, which is usually studied under the assumption of ideal surface terminations [16] and which is relevant for atmospheric processes like ice-nucleation. Another effect is related to the stability of these particles and consequently the release and transport of particles in the environment [17].

We have specifically studied the effect of intentionally added aluminum on the electrokinetic properties of kaolinite. As a simpler system, we have also studied the effect of added aluminum to a single crystal of sapphire to see how this would affect the IEP. The sapphire basal plane was chosen, because (i) this particular plane had been used in the above cited study on isostructural hematite [9] and (ii) a structurally and chemically very similar surface occurs on kaolinite particles. This part of the work was completed by a second harmonic generation (SHG) study as an alternative method to probe interfacial properties. The combined approach is the first direct comparison between electrokinetic and non-linear optics measurements on one mineral sample pre-treated in the same way. This is of relevance because no agreement exists as to whether the SHG signal is related to surface-potential [18] or zeta-potential [19], two different potentials in the electric double layer.

## Results and discussion

### Solubilities of relevant aluminum minerals

**Figure 1:**
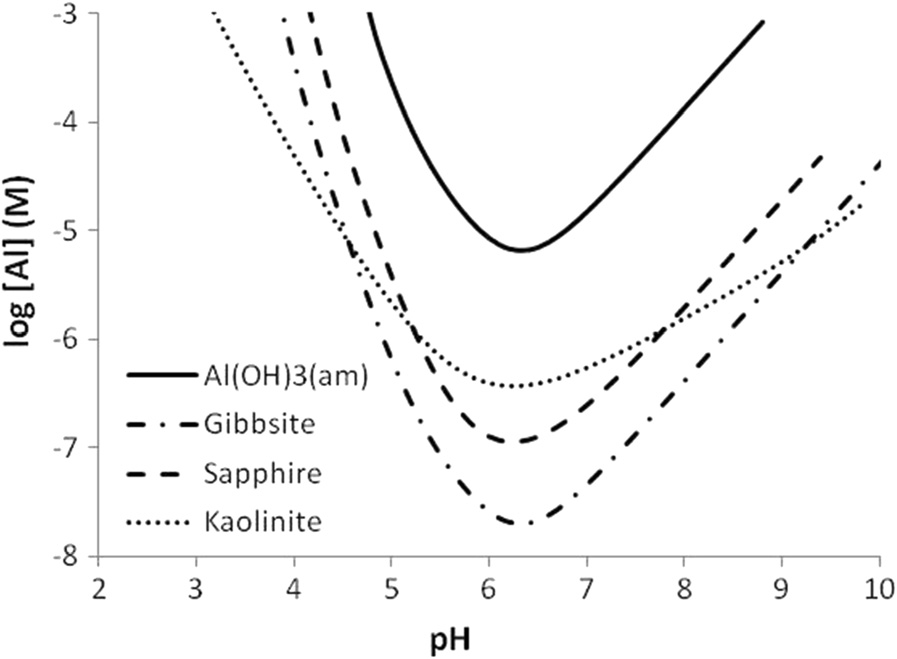
**Calculated solubility of some aluminum bearing minerals as a function of pH.** For kaolinite congruent dissolution is assumed, without considering potential precipitation of more stable solids.

Figure 1 shows the results of thermodynamic calculations to estimate the solubilities of relevant aluminum minerals. The thermodynamic data are given in Table 1. As described in the experimental part and also in the following section, the addition of aluminum was done at intermediate pH (for kaolinite) and at about pH 4 for sapphire-c.

**Table 1:** **Thermodynamic data used to produce the curves in Figure **1

**Reaction**	**Log K**
Al^3+^ + H_2_O = AlOH^2+^ + H^+^	-4.950
Al^3+^ + 2 H_2_O = Al(OH)_2_^+^ + 2 H^+^	-10.590
Al^3+^ + 3 H_2_O = Al(OH)_3_ + 3 H^+^	-16.420
Al^3+^ + 4 H_2_O = Al(OH)_4_^-^ + 4 H^+^	-22.870
Al(OH)_3(am)_: Al(OH)_3(s)_ + 3 H^+^ = Al^3+^ + 3 H_2_O	10.578
Gibbsite: Al(OH)_3(s)_ + 3 H^+^ = Al^3+^ + 3 H_2_O	7.738
Kaolinite: Al_2_Si_2_O_5_(OH)_4_ + 6H^+^ = 2 Al^3+^ + 2 H_4_SiO_4_ + H_2_O	6.472
Sapphire (α-Corundum): Al_2_O_3_ + 6 H^+^ = 2 Al^3+^ + 3 H_2_O	18.301

The calculations show that a total concentration of intentionally added aluminum of about 10 μM should not result in bulk solution precipitation of amorphous aluminum hydroxide. Amorphous aluminum hydroxide is expected to precipitate prior to the formation of gibbsite or other crystalline materials. Given that our experiments are short-term, it is reasonable to assume that no bulk precipitation of aluminum oxides, oxyhydroxides or hydroxides occurs. However, formation of surface precipitates cannot be excluded [20].

### Electrokinetic, SHG and AFM results for sapphire-c

**Figure 2:**
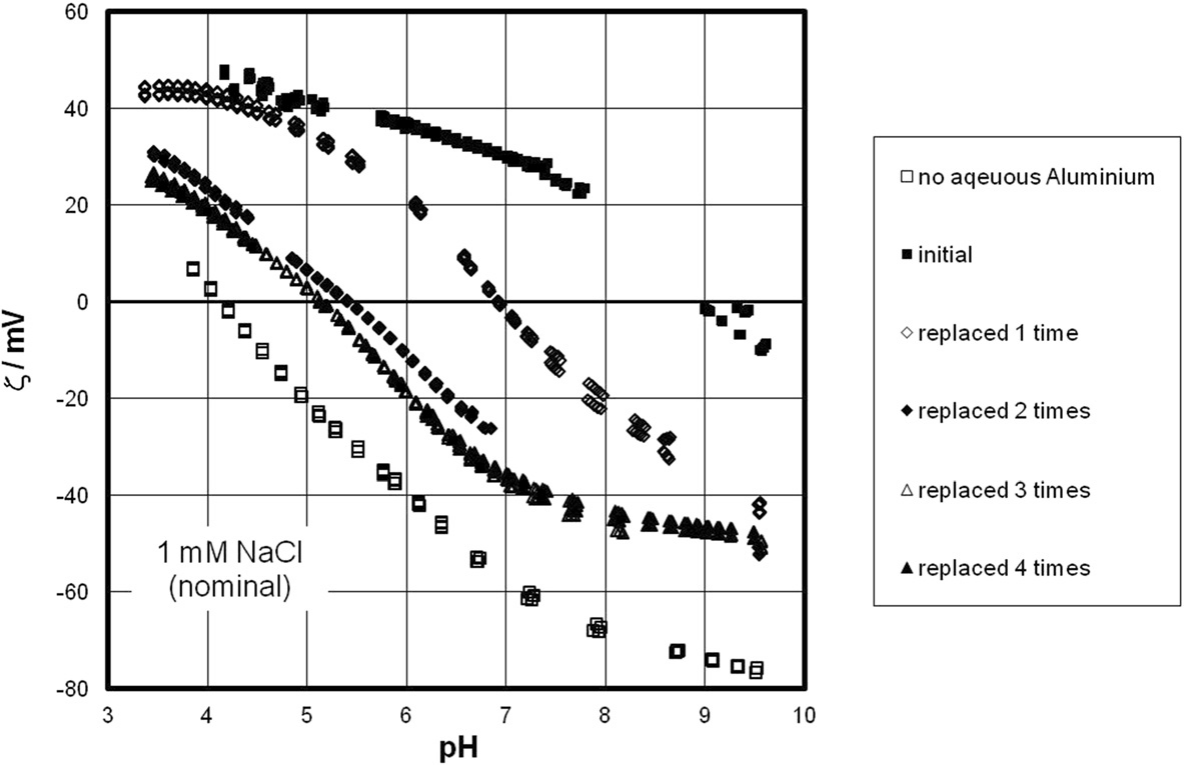
**Effect of aluminum on the zeta-potential of sapphire-c in 1 mM NaCl.** Open squares: in absence of added aluminum, full squares: after addition of aluminum (concentration of 3 μM), diamonds and triangles: after replacing the 500 ml solution from the previous experiment by 1 mM NaCl, and subsequent solution exchange.

Figure 2 shows the results of the streaming potential measurements with hydroxylated sapphire-c. In the absence of added aluminum the IEP is at about pH 4, in agreement with previous investigations [19,21,22]. This crystal plane ideally consists exclusively of doubly co-ordinated hydroxyls, which are not particularly reactive in the circumneutral pH range. We have previously interpreted the low IEP of sapphire-c that was found by us and many others by the “hydrophobic” properties of this surface. Subsequently the water structure on this surface has indeed been found to be weak compared to crystal planes that have more reactive hydroxyls [23]. This independent observation supports our previous assumption that sapphire-c behaves like hydrophobic surfaces which all exhibit generic pH dependent charging and have low IEPs [21,24,25]. The interfacial water profiles on other cuts of sapphire (and hematite) are much more pronounced, while the c-cuts exhibit water structuring similar to intrinsic hydrophobic surfaces as shown in the supplementary information to ref. [21]. On surfaces such as diamond, Teflon or gold preferential physical adsorption of hydroxide ions is a widely accepted mechanism [26]. The difference in affinity between proton and hydroxide ion for inert surfaces like Teflon (which do not bear any functional group that would cause pH-dependent behavior) causes the low isoelectric points. The accumulation of protons at the interface changes the sign of the measured electrokinetic potential at pH 4 (or below) implying that at pH 4 the surface densities of protons and hydroxide ions are equal (and not at pH 7, where they have equal concentrations in bulk solution). We will refer to this interpretation as “(preferential) hydroxide adsorption”. In the case of hydroxylated sapphire-c this unconventional hypothesis can explain the low IEP without resorting to impurity effects. Briefly, it has been previously shown that data sets for contact angle [19] and the water band at 3250 cm^-1^ [27] as a function of pH behave as predicted by the MUSIC model, while electrokinetic data show the IEP at pH 4. In other words the protonation/deprotonation reactions of surface hydroxyls suggest zero surface potential at pH 6, while the electrokinetic potential is strongly negative under the same conditions. The conventional surface complexation model cannot account for this. However, assuming that the (de)protonation of the surface hydroxyls is very weak and concomitant interfacial water ordering likewise is, it is possible to assume that interfacial species that occur on hydrophobic surfaces would also be attracted to the sapphire-c electrolyte interface. The situation is less ambiguous on silver halide surfaces [28,29]. Here, the surface potential is determined by the silver and halide ions resulting in surface potential that does not depend on pH, whereas the zeta-potential does. Clearly this can only be explained by a dual-charging mechanism, with silver and halide ions adsorbing in the surface plane while protons and hydroxide ions adsorb further away from the surface plane. The latter involves reactions with the water layer and not with surface sites. On sapphire-c this dual-charging was previously postulated and can explain interfacial data on sapphire-c without involving impurities. We note that specific treatment of sapphire-c and in particular plasma cleaning increases the IEP. Our samples were not plasma cleaned and the results in Figure 2 agree with previous data [3,19,21].

In the zeta-potential measurements on sapphire-c the first addition of aluminum to sapphire-c was done at low pH and causes the zeta-potential to rise to positive values whereby the IEP shifts from pH 4 to nearly pH 9. The latter is within the range of IEPs of aluminum oxide particles [1,2]. After Al addition and the subsequent titration the solution was replaced by 1 mM NaCl solution. Then a first measurement was taken at pH about 6. Subsequently the pH was increased again and a downward titration was carried out. The IEP dropped to pH 7, which we assume is due to (partial) desorption of previously adsorbed aluminum. A second replacement of the solution causes the IEP to further lower to about pH 5.5, and the third replacement shifts the IEP to pH 5. Subsequent solution replacements do not further lower the IEP. In all the replacement solutions an initial measurement was made at approximately pH 6 prior to increasing the pH. This series of experiments shows that (i) dissolved aluminum interacts with sapphire-c, (ii) that the IEP can be decreased by solution replacement (in our interpretation a significant part of the adsorbed aluminum can be desorbed), and (iii) that some aluminum has an irreversible influence on the zeta-potential. The important point is that interaction with dissolved aluminum may strongly affect measured IEPs, which we think might play a role in reported IEPs for kaolinite, for example.

We verified the electrokinetic observations by concomitant SHG experiments and AFM controls.

**Figure 3:**
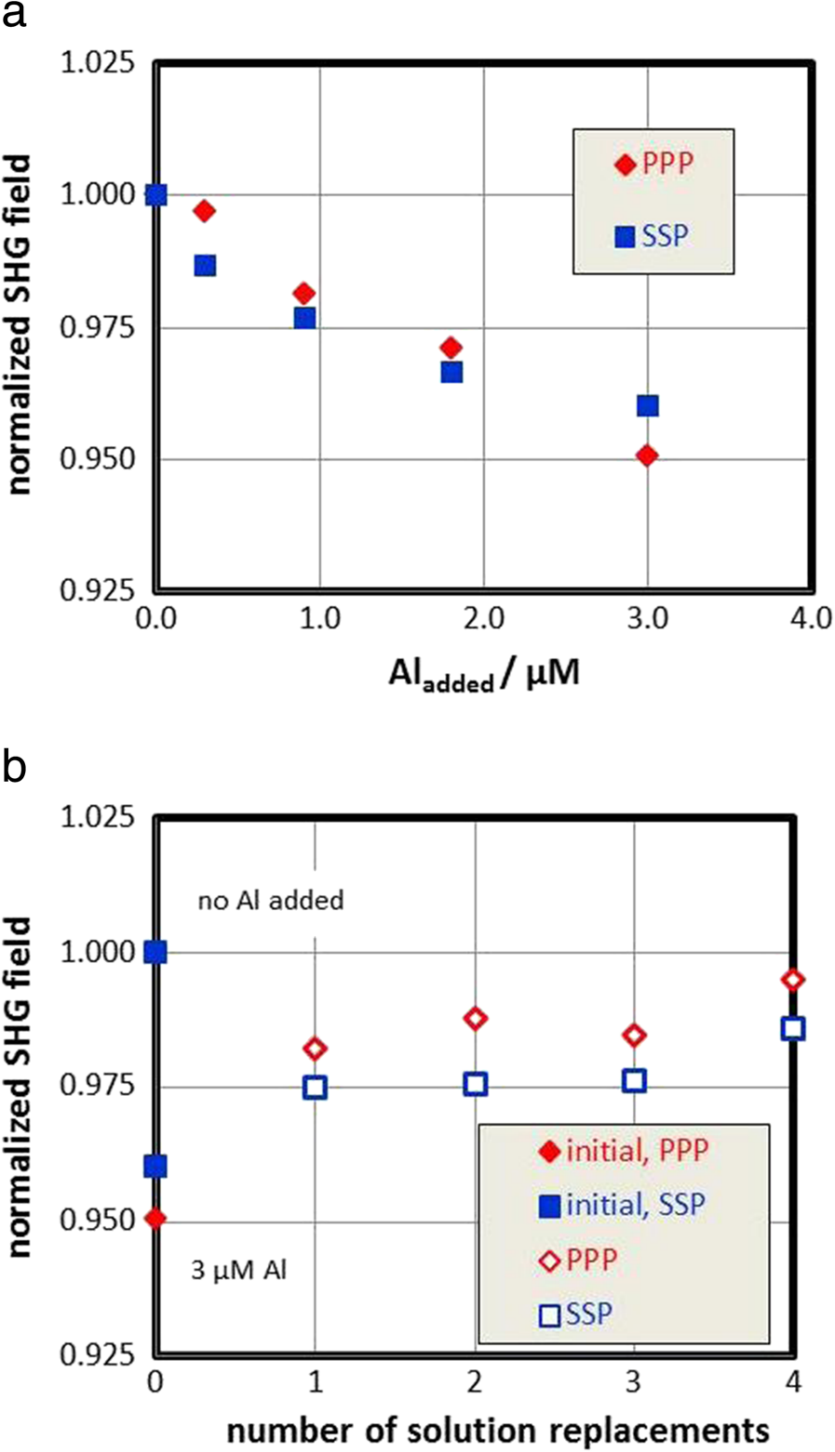
**Effect of aluminium on SHG signals. a** Effect of the addition of aluminum solution at pH 6 on the normalized SHG field in 1 mM NaCl for sapphire**-c**. Final concentration is 3 μM to mimic the streaming potential measurements shown in Figure 2. PPP and SSP are the two polarization combinations described in the experimental part. **b**. Effect of replacing the 3 μM aluminum solution containing 1 mM NaCl by fresh 1 mM NaCl solutions on SHG results for sapphire-c at pH 6. The initial values are from Figure 3a. Normalization of the raw data was done with respect to the absence of aluminum.

Figure 3 summarizes the results from SHG experiments carried out at pH 6. In a first series aluminum solution was stepwise added to 500 ml of 1 mM NaCl solution. The results are shown in Figure 3a. Clearly the addition of aluminum causes a decrease of the SHG field, which is a consequence of changes in the water ordering at the interface. The decrease is accompanied by a change in the interfacial zeta potential, in our case either an increase in positive or a decrease in negative zeta-potential with concomitant reversal of the sign [30]. The SHG results imply that the orientation of the water dipoles at the interface is affected by the adsorbed aluminum. In the present case the orientation of water dipoles prior to the addition of aluminum is such that the electrostatics will attract the positive part of the dipoles to the surface. This attraction is lowered, when the surface becomes less negatively charged, weakening the signal. A charge reversal cannot be inferred from the SHG data. The trend agrees with the one observed by Fitts et al. [30] for bare sapphire single crystals. In that case the SHG signal decreases with decreasing pH, i.e. a lowering of the negative potential occurs above the “point of zero charge” and an increase of the positive potential occurs below the “point of zero charge”. As a conclusion the SHG results show the same trends as the measured zeta-potentials.Figure 3b shows the results obtained when replacing the solution in the same way as in the streaming potential measurements (Figure 2). After reaching the same aluminum concentration as used in the first series in the streaming potential experiments with added aluminum, the solution was replaced 4 times by fresh 1 mM NaCl solutions.

Data are referenced to the SHG field measured in the absence of aluminum. The replacement of solution causes a re-increase in the SHG signal and a relaxation towards the aluminum-free system, which was also observed in the zeta-potential measurements.

To directly relate the data to each other, we scaled both data sets by the formula

(1) $$q_{i}=1–\left(m_{i}-m_{o}\right)/{\left(m_{3µM}-m_{o}\right)}_{\max}$$

where the subscript “i” denotes the number of solution replacement, q_i_ is the scaled quantity (i.e. either from zeta-potential or SHG measurements), m_i_ is the measurement value, m_o_ is the measurement value in the absence of aluminum and (m_3μM_-m_o_)_max_ is the difference between the measurement in the presence of 3 μM aluminum solution and in the absence of aluminum.

**Figure 4:**
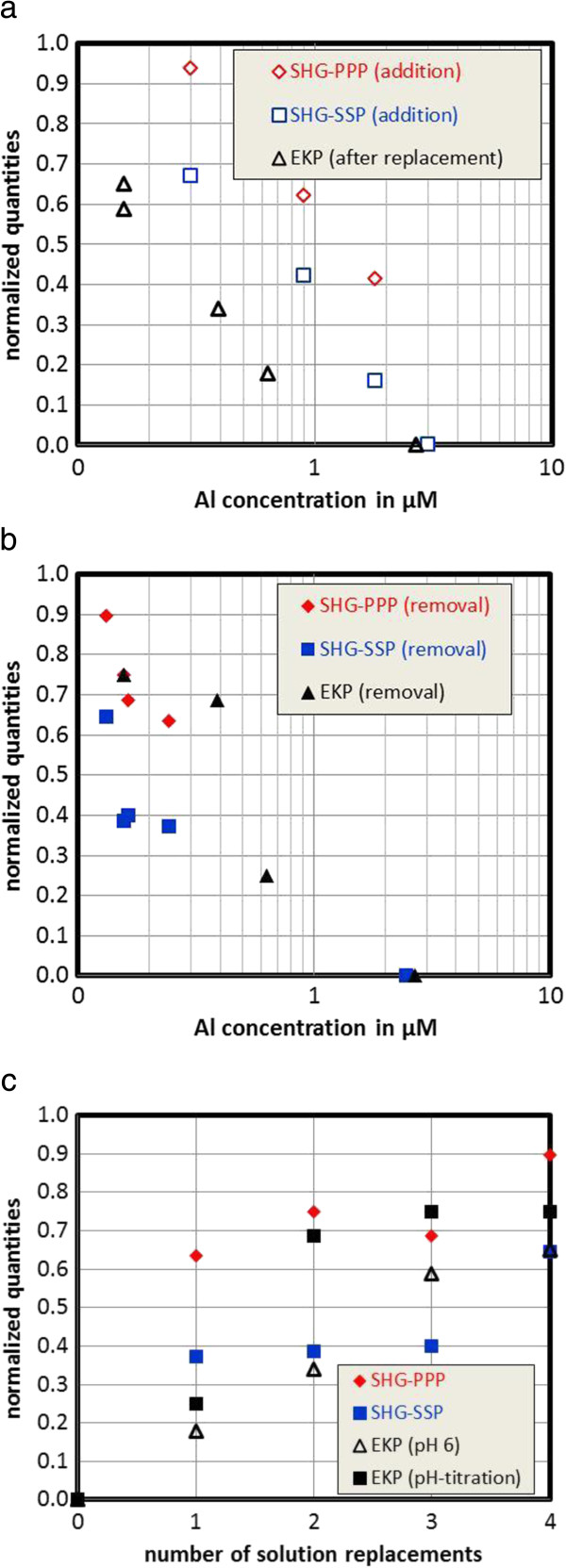
**Scaled quantities from zeta-potential measurements and SHG for the different solution replacements; a and b: data plotted as a function of measured Al-concentration.** In Figure a the SHG data were collected after the step-wise addition of Al; the EKP (electrokinetic potential) data pertain to the first measurement made after replacing the solution (i.e. prior to increasing the pH). In Figure b data pertain to measurements involving removal (i.e. replacement of the solution). Figure 4c shows the replacement data as a function of the number of solution replacement.

The outcome is summarized in Figure 4. Figure 4a compares the SHG results from the addition sequence (Figure 3a) with zeta-potentials measured directly after solution replacement as a function of measured Al-concentrations. Figure 4b compares the scaled quantities from the solution exchange sequences from both sets of experiments as a function of measured Al- concentration and Figure 4c as a function of the solution replacements. The trends in the data agree quite well. We note that it is not clear which interfacial potential is probed by SHG. The data of Fitts et al. [30] for sapphire-c showed a cross-over point at various sodium nitrate concentrations which coincides with the IEP obtained for that surface by streaming potential [19,21]. The present direct comparison might confirm this, although the scaling procedure does not allow an ultimate conclusion on this important issue.

**Figure 5:**
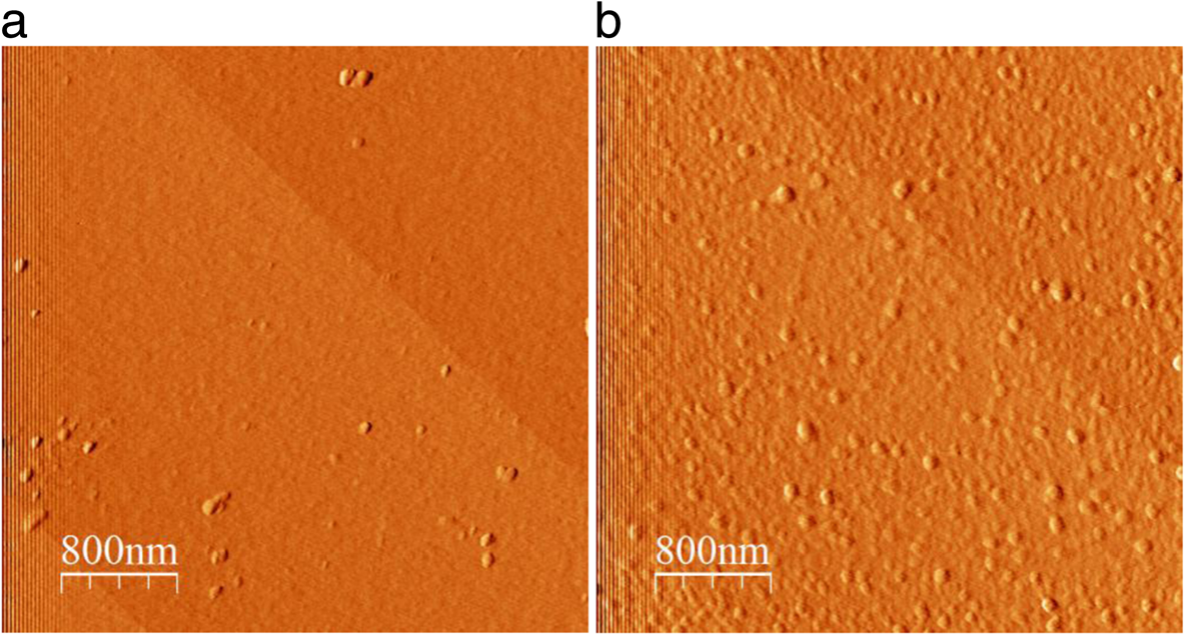
**AFM pictures of the c-plane of sapphire on the prism used for the SHG measurements.** At spots that were exposed to solution not containing Al (a) and one spot that had been exposed to 3 μM aluminum and subsequently rinsed with 1 mM NaCl solutions several times (b).

The conditions for the experiments can be related to the calculated equilibrium solubility of sapphire at pH 6, which is slightly lower than that of the amorphous hydroxide. From Figure 1 no amorphous hydroxide precipitate in the bulk solution would be expected. However, we inferred from the electrokinetic results that only a fraction of Al that must have reacted with sapphire can be easily removed. In the simplest phenomenological interpretation, the removable fraction would correspond to adsorbed aluminum, whereas the remaining fraction on the surface would be in the form of a surface precipitate. To verify the existence of a precipitate, the prism from the SHG measurements was studied by AFM after the last solution replacement. Based on Figure 5 the surface shows some morphological change, which may be attributed to a surface precipitate, which in turn could represent the irreversibly bound aluminum. Figure 5a shows the initial surface, which has been in contact with aluminum free solution, and is rather smooth, though not perfect. In contrast, Figure 5b, taken after the experiments, clearly exhibits many irregularities. We stress that we do not have an ultimate proof for our interpretation, but both the AFM images and the lack of full reversibility would seem to support our simple picture. The features found on the surface are disk-like overgrowths (50 to 100 nm in width and 2 to 3 nm in height). Such features are reminiscent of gibbsite platelets [13]. The IEP of gibbsite is usually expected to be around pH 10 [13]. CTR measurements on sapphire-c have provided evidence of formation of a gibbsite-like surface [31]. However, our interpretation remains circumstantial and more work is required to resolve this issue.

Overall our interpretation would suggest a twofold interaction of dissolved aluminum with sapphire-c: the formation of adatoms similar to those previously reported for hematite [9] and surface precipitation of some aluminum bearing phase. The ease of removal of the adatoms would agree with similar, more direct observations by Eggleston et al. [9].

### Electrokinetic and surface complexation model results for kaolinite

**Figure 6:**
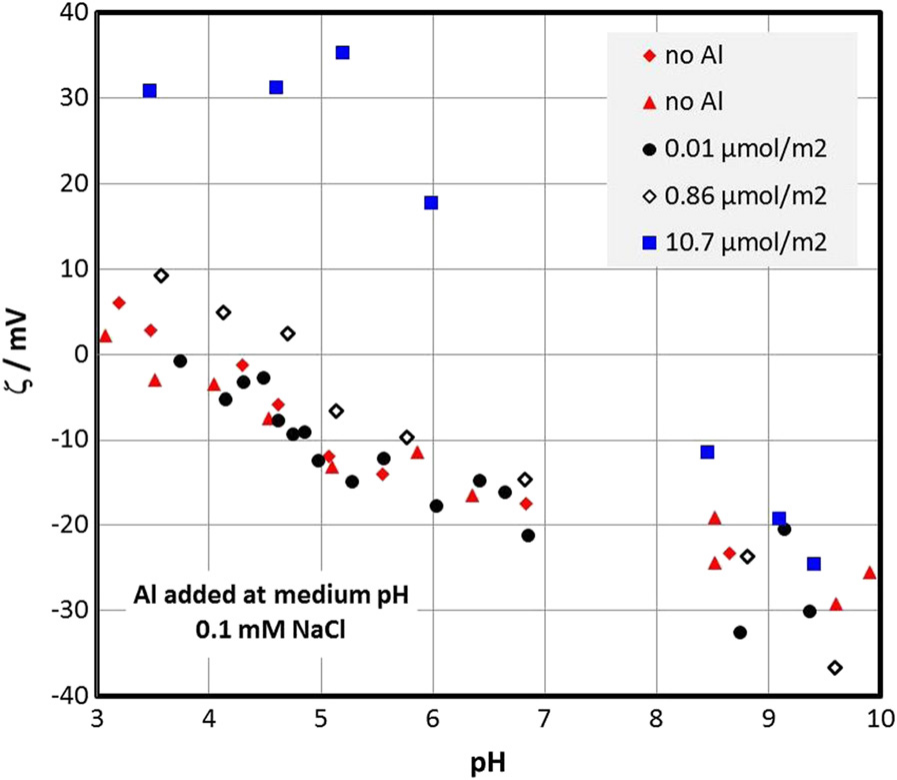
**Zeta-potential of kaolinite as a function of pH in 0.1 mM NaCl with and without added aluminum.** Total aluminum is given as maximum coverage that could possibly be achieved by the intentionally added aluminum.

For KGa-2 kaolinite when no Al is added (filled triangles and diamond in Figure 6) the IEP is found at pH 3.5, well within the range reported for kaolinite in general and for KGa-2 in particular [1,2] (see background section). Addition of Al solution at intermediate pH (filled circles, squares and open circles in Figure 6) shifts the IEP to higher pH values. Clearly the addition of Al has a profound effect on the electrokinetics of kaolinite. Our results agree with previous measurements by Hall [32]. Direct comparison with our data is not possible, since the kaolinite surface area is not given, but because the particle size is above 1 μm, we infer that Hall’s particles were larger than ours, which would explain why 30 mV were observed at the lowest Al concentration studied by Hall. In our system such high values were obtained with the highest Al-concentration studies.

For kaolinite the overall elemental composition has been found to affect the IEP and in particular increased Al-content was found to increase the IEP [33]. Al adatoms would therefore be one realistic cause among others for variable IEPs of one specific sample. Other minerals could be likewise affected by adsorption of solutes that can be formed from the mineral. It has been shown that Al_13_O_4_(OH)_24_^7+^ may form during laboratory synthesis of gibbsite [34]. It has been used to stabilize gibbsite particles against aggregation [35] and it was hypothesized that its presence during synthesis may subsequently affect the surface properties of gibbsite [13]. This Keggin-ion has also been shown to adsorb to sapphire-c and to affect the surface properties accordingly [36]. The present study would suggest that monomeric aluminum can also be adsorbed to gibbsite. Overall various properties can be generated depending on the speciation of dissolved aluminum during gibbsite synthesis. The occurrence and extent of the effect would depend on the pH to which the sorbent was exposed and for how long it was exposed. Furthermore, it would be important to know if the solution was replaced or if a pH-dependence study was started from some extreme pH. Finally the solid to liquid ratio will be crucial.

Figure 6 shows a clear shift of the IEP with increasing aluminum concentration. The presence of dissolved aluminum will therefore cause a higher IEP compared to a system where no aluminum was added. The start of an electrokinetic experiment at low pH may cause release of aluminum due to dissolution (Figure 1). The dissolved aluminum can then adsorb to kaolinite when the pH is increased (as observed on sapphire-c). Such phenomena will not necessarily occur if every sample is separately prepared from near neutral stock suspensions for example. Another scenario is an acid wash pre-treatment that involves either settling or some other kind of solid liquid separation prior to washing with water or electrolyte solution to remove excess acid. If not all of the aluminum that was potentially dissolved during the acid-wash can be separated during the first wash, the remaining aluminum may later adsorb to kaolinite (due to changing pH) and affect the IEP that is obtained. In the case of clays dissolved aluminum may also be fixed on the ion-exchange sites of the particles and thus remain with the solids. This has been discussed by Schindler et al. [37]. The situation with the clays in general and with kaolinite in particular is complex because the two faces of kaolinite have different charging behavior [38] and the edges differ from the faces [39]. In principle dissolved aluminum might interact with all of these kaolinite surfaces. These possible scenarios make it mandatory for any study on clay minerals to describe in detail how the experiments were carried out and whether and how the sorbent samples were pre-treated. Otherwise it is impossible to exclude any of the possible scenarios depicted above.

**Figure 7:**
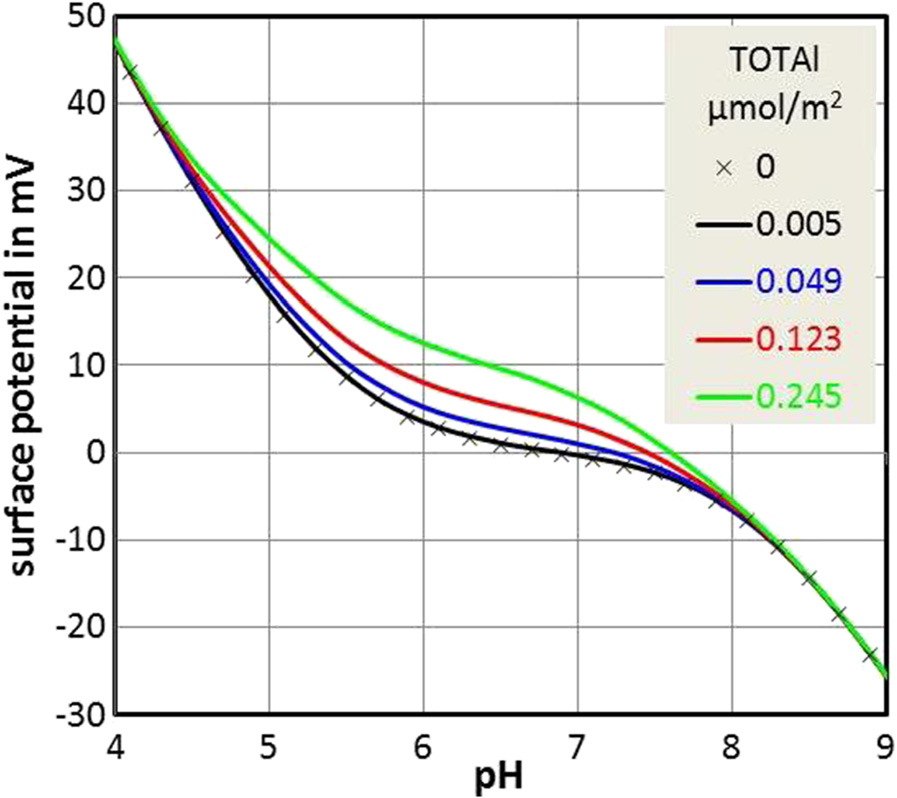
**Results of surface complexation calculations for aluminum sorption onto kaolinite.** The calculations are based on the model by Schindler et al. [37] and pertain to 1 M NaClO_4_ media and a constant capacitance model.

Figure 7 shows calculations using a surface complexation model (details are given in the Methods section, the parameters are given in Table 2). The original model had been derived for divalent metal adsorption on kaolinite [37]. The reported parameters were used to construct linear free energy relationships (based on first hydrolysis constants) from which parameters can be extrapolated to dissolved aluminum. We plot the calculated surface potential (that is different from the zeta-potential) as a function of pH for different amounts of Al added to the system. Clearly the presence of aluminum generates more positive surface potentials, in agreement with the experimental electrokinetic data (Figure 6).According to the generic surface complexation model, surface complex (or adatom) formation of aluminum starts at pH 4.5. No visible effect is obtained with the lowest aluminum concentration in Figure 6. Exposure to higher concentrations causes changes in the surface potential of below 10 mV, comparable to the experimental zeta-potential data. At high pH the curves merge in both the experimental data and the calculations.

**Table 2:** **Surface complexation parameters used for the calculations based on Schindler et al. **[37]

**Species**	**log K**
Al(OH)^2+^	-5.47
Al(OH)_2_^+^	-10.28
Al(OH)_3_	-16.08
Al(OH)_4_^-^	-23.66
SOH_2_^+^	4.27
SO^-^	-9.18
SONa	-9.84
SOAl^2+^	-0.54
(SO)_2_Al^+^	-2.41

C = 2.2 F/m^2^. Basis: Na^+^, Al^3+^, H^+^, SOH, I = 1 M NaClO_4_. Ion-exchange was omitted because of the high ionic strength. Site density (TOTSOH) was 39.9 mmol/kg.

It is not a priori clear where the aluminum adsorbs on kaolinite. One obvious candidate is the “gibbsite” face of kaolinite. This crystal face is structurally similar and with respect to its protonation behavior more or less identical to the hydroxylated sapphire-c plane (and the basal plane of gibbsite), which does interact with dissolved aluminum (Figure 2). However, it cannot be excluded that interaction occurs with the silica face of kaolinite or with edge sites. It has been shown by Gupta et al. [40] that the charge of the basal planes of kaolinite is very small compared to the edges. For their kaolinite they obtain an IEP of about 4.5 and the charge is entirely dominated by the edges. We expect that adsorption of Al to any of the faces would affect the overall zeta-potential (i.e. none of them is sufficiently positive to be excluded).

While the accidental presence of aluminum due to exposure of kaolinite to harsh conditions (i.e. high or low pH) and subsequent titration of the batch from those values involves the same problems as discussed for sapphire-c (such as duration of exposure), the aluminum-adsorption process is more than likely pH-dependent. Determination of such pH dependence is notoriously difficult, since any measured aqueous aluminum concentration can be due to dissolution/precipitation and adsorption/desorption processes.

The surface complexation model was also used to check our interpretation that adsorbed aluminum can be removed from the surface of sapphire by solution replacement only. The calculations show that due to the high solution volume to surface area ratio for the streaming potential and SHG measurements, the removal procedure rapidly generates conditions, under which the surface potential is not affected by the aluminum remaining. This is quite different from situations with low solution volume to solid ratios where similar calculations show that it is difficult to remove adsorbed ions by solution replacement [41].

### Experimental

All solutions were freshly prepared from MilliQ water (18.2 MΩ · cm). Purified argon was used to minimize intrusion of carbonate into the solutions and measurement systems in the case of the electrokinetic experiments.

Aluminum stock solutions were prepared from analytical grade AlCl_3_. The concentration of the stock solution was checked by ICP-OES.

The c-plane samples used for the streaming potential measurements were obtained from TBL Kelpin (now MaTeck, Jülich). They were 10 mm by 20 mm and 0.5 mm thick, polished on one side. Before use the samples were subject to the standard cleaning protocol established by Rabung et al. [42]: samples were soaked in acetone overnight, subsequently washed with ethanol, then soaked in ethanol (2 hours), washed with Milli-Q water and finally soaked in Milli-Q water (1 hour). This eliminates organic carbon contamination and limits inorganic contaminations to a minimum [42].

The streaming potential measurements were done with the SurPass Electrokinetic Analyzer (Anton Paar) at room temperature. The set-up has been previously described in much detail [19,43]. Prior to each measurement series the pH and conductivity electrodes were calibrated. The electrodes were found to be very stable over the duration of the experiments as verified against buffers and standards after the respective experiments. The single crystals were mounted on the stamps of the gap cell and the gap was optimized until pressure ramps (flow rate against pressure) were coinciding from both flow directions and the gap height was within the recommended range (typically 100 μm).

The first measurement was done in an electrolyte solution (1 mM NaCl). Purified Ar flowed over the electrolyte solution at all times. Then the pH was raised to about 9.5 by adding NaOH (0.1 M) and subsequently a titration with acid was started. The IEP of the bare surface at pH about 4 agrees with previous studies [19,21].

To study the effect of dissolved aluminum on zeta-potential and IEP, aluminum stock solution was added at about pH 4 to yield a total aluminum concentration of about 3 μM. Then the pH was increased to 9 by adding NaOH (0.1 M). During this process, no separate solid phase was visually detectable in the liquid phase over the full series of titrations. The system was then titrated down to pH 3 and the zeta-potential measured as a function of pH. Subsequent experiments were carried out to investigate the effect of solution exchange at low pH (i.e. the decrease of dissolved aluminum) to check to what extent the effects initially observed could be reversed. At the end of a given titration the “empty” function of the control software was used to remove solution from the system. The “empty” process never removes all solution from the apparatus and it was not attempted to achieve complete removal which would have required dismantling the set-up, rinsing with solution or restarting an experiment with new conditions (in terms of gap height for example). Instead a fresh 1 mM NaCl solution was used to refill the system and single point measurements in that solution were carried out. Subsequently, the pH was adjusted to approximately 9.5 by adding NaOH (0.1 M) solution and a new downward titration with zeta-potential measurement was started. This was repeated until two subsequent titration-data coincided.

Selected SHG experiments were carried out using sapphire prisms, exposing part of the prism’s basal plane to the solution. The prisms had been treated in the same way as the single crystals used in the streaming potential measurements. The set-up for the SHG experiments is described in detail below. SHG experiments were carried out at 18°C. No significant change was observed when the temperature changed by ±2°C. The prisms for the SHG experiments were obtained from Victor Kyburz AG, Safnern, Switzerland.

**Figure 8:**
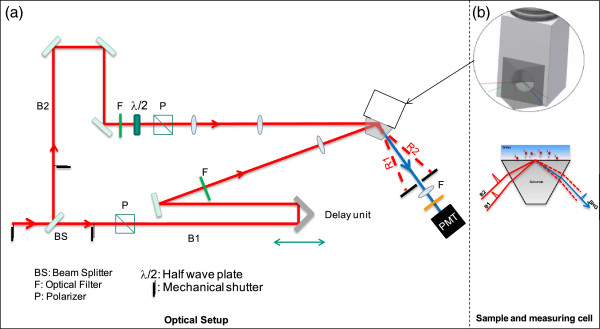
**Set-up for the SHG measurements (a) Drawing of the TR-SHG used in this work. (b)** Sketch of the measuring cell and sample geometry.

The SHG experiments were conducted using a femto-second laser system (Solstice from Spectra Physics) with a fundamental beam of 800 nm wavelength, 3.5 mJ pulse energy, ~80 fs pulse width, 1 kHz repetition rate, and a diameter of 1 mm at the interface. The experiments were performed in total internal reflection (TIR) geometry. This geometry provides an additional enhancement in the SHG signal if the incident beams are close to the critical angle. Instead of having one fundamental beam incident on the interface, two incident beams at two different angles were allowed to overlap in space and time on the sample surface in a similar way as in sum frequency generation (SFG) experiments. The difference here is that both incident beams have fixed equal wavelengths. A photomultiplier (PMT) was used to collect the SHG signals.A Time-Resolved SHG (TR-SHG) in TIR geometry (Figure 8) was used to probe the water structure near the solid surface. The fundamental beam is split in two paths (B1 and B2) using a 50% beam splitter. The two split wave fronts of each pulse are allowed to overlap in space and time at the interface. The equality of the optical paths is assured using a delay unit placed in the path of B1. B2 is allowed to be either S-polarized (perpendicular to the plane of incidence) or P-polarized (parallel to the plane of incidence). This is achieved by a half-wave-plate followed by a cube polarizer. B1 is constantly P-polarized and controlled by a cube polarizer. The generated signal is collected using a PMT placed beyond an optical system, which includes appropriate filters and polarization analyzers. The background signals which may arise from different optics and which may accompany the split fundamental beams are of no relevance because they follow different paths (after reflection) from that of the signal generated at the interface (signal to be probed). This is one advantage of using TIR-SHG geometry. Another advantage is the high field intensity at the interface, which enhances the generated signal.

The substrate of interest is pressed against a Teflon cell with an opening of 8 mm diameter. The hypotenuse of the prism (c-cut) faces the opening in the Teflon cell. The solution inside the cell is constantly cycled between the cell and a 500 ml flask, which contains the major part of the solution. Contact to the atmosphere was thus minimized. The temperatures of the sample top, sample bottom and water inside the cell are measured using four-wire-Pt100 elements. The incident angle of B1 on the interface is set to an angle slightly higher than the critical angle of TIR for the sapphire/water interface. The incident angle for B2 is set at angular distance of 17° from B1, which is higher than the critical angle.

The SHG response relates to the overall arrangements of dipole moments. Two polarization combinations are being measured in the present study. The PPP polarization combination corresponds to P-SHG, P-B2 and P-B1. The SSP polarization combination corresponds to S-SHG, S-B2 and P-B1. These two combinations should be sufficient to describe this system which has C_2v_ symmetry [44]. The higher the signal of either SSP or PPP the higher is the ordering of the molecules while the ratio of SSP/PPP determines the average orientation of the interfacial molecules.

The SHG experiments were conducted at pH 5.8. Aluminum was added stepwise to the solution to change the dissolved aluminum concentration up to 3 μM. All solution compositions correspond as closely as possible to those used in the previous zeta-potential measurements. The results from the latter conveniently allowed a definition of suitable experimental conditions. After reaching the maximum aluminum concentration desired, the solution was replaced in the same way as in the streaming potential experiments. The samples used for the SHG experiments were examined by AFM corresponding to conditions after the cleaning step (contact with water) and after the SHG experiments (contact with Al-bearing solutions).

Dissolved aluminum concentrations in solution were determined by ICP-MS. This was done for solutions used in the streaming potential measurements and the SHG experiments. Due to the low amount of surface area exposed to rather large volumes of solution (streaming potential 4 cm^2^, SHG less than 1 cm^2^ exposed to 500 ml of solution) it was not possible to quantify uptake. Basically the amount removed by the sapphire-c surfaces is negligible compared to the total amount. The same is true for the kaolinite experiments described hereafter, where no Al-determination was carried out.

The interaction of dissolved alumina with kaolinite was studied using traditional electrokinetic measurements (Brookhaven PALS). For these experiments the international standard sample KGa-2 of the Clay Mineral Society Source Clay Repository was used. KGa-2 is a natural clay containing 96 wt.% disordered kaolinite (Hinckley index 0.37 ± 0.05), 2 – 3 wt.% anatase, 1 wt.% crandalite and traces of mica, illite, halloysite and amorphous iron hydroxide [45,46]. Details about the origin of the sample have been published elsewhere [47,48]. The kaolinite sample was not purified and treated prior to the measurements. The external surface area was derived by the Brunauer-Emmett-Teller (BET) method [49], using 5 points of the N_2_ adsorption isotherms collected for the sample. After outgassing at 80°C under N_2_ gas flow, 19 replicate nitrogen adsorption isotherms were measured employing a Micromeritics Gemini II-2375 surface area analyzer. The BET specific surface area of KGa-2 was found to be 18.7 ± 1.9 m^2^ g^-1^, which agrees with previous measurements by Metz and Ganor [50]. Relatively high BET-surface area results for KGa-2 measured by Dogan et al. [51,52] (21.7 ± 0.3 m^2^ g^-1^), Madsen [53] (24 m^2^ g^-1^) and van Olphen and Fripiat [47] (20.0 to 24.1 m^2^ g^-1^) result from their outgassing techniques in vacuo and relatively high temperatures, i.e. 130 to 200°C, as demonstrated for KGa-2 and other standard clay samples [46,54]. Applying the glycerol saturation method, Madsen [53] determined 25 ± 4 m^2^ g^-1^ as the specific total surface area of KGa-2. Surface properties, chemical composition, cation exchange capacity, morphological and crystallographic properties are given in the literature [46-57].

The kaolinite suspensions were prepared for the zeta potential measurements by transferring a known amount of dry kaolinite powder (~0.0052 to 0.0520 g) into a 200 mL beaker that contained 100 mL of Milli Q water and pre-determined aliquots of 100 mM NaCl to yield ionic strength of 0.1 mM. Initial experiments had been carried out to determine suitable solid/solution ratios and ionic strength. The kaolinite suspensions that were intended for studying the effect of added aluminum were spiked with 10 mM Al(III) to yield final concentrations in the range Al(III) 0.1 - 10 μM. The batch suspensions were always hydroxylated by stirring for 1 h under continuous Ar purging. The pH of the hydroxylated kaolinite suspension was around 5.08 - 5.99 (depending on the solid concentration). Prior to each experiment, the pH of the kaolinite suspensions was adjusted to pH ~9 by adding from a 0.428 M NaOH solution. Subsequently, the pH was gradually decreased at 0.5 pH intervals by the addition of either 0.420 M or 0.200 M HCl. At each point an aliquot of the kaolinite suspension, typically 2 mL, was slowly transferred into a measurement cell under Ar. The electrophoretic mobility was measured under an argon cushion. An average of at least five independent measurements was taken for each sample. A controversy exists about the use of Smoluchowsky formula to convert electrophoretic mobility data into zeta potential, regardless of the particle morphology. Morrison [58] has shown that in the limits of κL > > 1 (κ inverse of Debye length, and L the characteristic length of the particle) the Smoluchowsky formula is applicable for particles irrespective of their morphologies. Sutheimer et al. [59] showed that KGa-2 kaolinite particles exhibit hexagonal shapes with irregular basal planes. The mean hydrodynamic diameter of the kaolinite was around 450 nm. Under these conditions, we invoked the software inherent Smoluchowsky function to calculate zeta potential from the electrophoretic mobility values, U, according to Equation 2

(2) $$\zeta =\frac{\mathrm{\eta U}}{\epsilon_{0}\epsilon_{1}}$$

where η, ϵ_o_ and ϵ_1_ have their usual meanings.

The calibration of the set-up was carried out using latex 707 nm standards according to the manufacturer instructions.

Atomic force microscopy (AFM) measurements were performed on a Bruker Dimension 3100 AFM employing a Nanoscope IV controller in contact mode on the dry sample surface using a Bruker SNL10 probe (nominal spring constant 0.06 N/m, nominal tip radius 2 nm).

## Conclusions

The experimental data collected in the present study show that interfacial reactions can be strongly affected by side reactions that are not foreseen in the usual design of experiments with pure samples. The aluminum bearing systems discussed in detail are all prone to the adsorption of dissolved aluminum that was added to the system on purpose, as in the present study, but may also originate from exposure of the system to extreme conditions. In general we have shown that such reactions present one possibility (amongst others) to explain a wide range of varying reported properties (such as IEPs) for nominally identical systems.

In the case of the single crystal sapphire the addition of aluminum exclusively affected the basal plane behavior, since no other surface is exposed. The observations concur with previous ones concerning the presence of ferric iron on isostructural hematite [9], though on hematite the reactions were observed at much lower pH. This is possible due to lower hematite solubility compared to sapphire and expected because of stronger hydrolysis of dissolved ferric iron compared to aluminum. SHG experiments confirmed the results from the zeta-potential measurements. The trends in SHG and zeta-potential data showed good quantitative agreement, suggesting that SHG probes interfacial properties similar to those probed in electrokinetic experiments. In our interpretation part of the added aluminum is present as adatoms that can be removed by rinsing with water, while another part may have precipitated on the surface, and is not easy to remove. Interestingly, in a recent study on the gibbsite basal plane, rather unexpected interaction with simple ions was directly observed [60]. Alkaline and alkaline earth ions were found to adsorb on gibbsite basal planes. In particular, ordered structures of Ca were found. Furthermore, it was found that these structures could be easily removed by washing (Siretanu and Mugele, personal communication). This further supports our interpretation that a fraction of adsorbed aluminum can be easily desorbed. In the case of kaolinite particles it is not possible to assign the observations exclusively to interactions of the dissolved aluminum with (i) edge, (ii) silanol or (iii) aluminol faces. However, the interaction with a negatively charged basal plane should have the most pronounced effect.

Our results suggest that reported data may have been compromised by very subtle details of sample pre-treatment and procedures, details which are not usually reported in the literature.

Future work might focus on the influence of the different surface properties on the ad/desorption of water on clay minerals and their impact on natural processes, like ice nucleation for instance, which is currently studied on ideal surfaces [16,61], while our survey based on the compilations by Kosmulski [1,2] shows that natural samples have widely varying properties. The SHG data imply changes in the water dipole orientation caused by the adsorbed aluminum, which in turn will have repercussions on processes like ice nucleation. Likewise the presence of aluminum may cause unwarranted competition on clay-based sorbent. In the case of montmorillonite, it has been shown that dissolved aluminum shows all the experimental and modeling features of metal ion adsorption that would be expected [62].

## Methods

### Thermodynamic calculations

#### ***Solubilities***

Calculations to estimate the solubility of relevant aluminum-bearing minerals were carried out using Phreeqc [63] and the Thermodem data base [64]. The full set of thermodynamic data is given in Table 1.

#### ***Surface complexation modelling***

Illustrative surface complexation calculations were carried out for the kaolinite system. The surface complexation model established by Schindler et al. [37] for the adsorption of Cd, Cu, and Pb on kaolinite was used to establish a linear free energy relationship to estimate adsorption parameters for dissolved aluminum on kaolinite. The original surface complexation model was a constant capacitance model, which allows calculation of surface potentials as a function of pH, but not zeta-potentials. Nevertheless, the trends in the surface potential from the calculations can be compared to the measurements. Zeta-potentials should be smaller in magnitude than surface potentials.

The surface complexation model was not established by Schindler et al. [37] based on the real point of zero charge of their kaolinite sample. They used potentiometric titrations, and as discussed in the introduction, PZNPCs of kaolinite are typically higher than IEPs, which in turn are more representative of the absolute overall charge of the particles. The PZNPC was established at about pH 6.8. Schindler et al. [37] give their parameters for infinite dilution but specify how they corrected for ionic strength. We used the same correction to obtain all parameters at 1 M. We then plotted the available log K values for the two kinds of surface complexes (monodentate and bidentate) for the available ions as a function of the respective first hydrolysis constant, resulting in two linear free energy relationships (LFERs). From the two LFERs Al-surface complexation constants were obtained to describe the formation of adatoms. The Al-hydrolysis constants were taken from Baes and Mesmer [65], since Schindler et al. also took the auxiliary data from that compilation. The ionic strength corrections were also made using the parameters given in Baes and Mesmer. The final calculations were done for a 1 M sodium perchlorate background, because ion exchange is not relevant for such a high background electrolyte concentration. Consequently no additional parameter for aluminum-ion exchange needs to be estimated. Interestingly Schindler et al. [37] in their paper discuss the possibility that aluminum is present on exchanger sites at the onset of the experiments, and add that subsequent hydrolysis of Al^3+^may have impacted their potentiometric titrations in unwarranted ways. Still the available model parameters from this study are comprehensive and self-consistent.

Calculations were done with a modified version of FITEQL2.0 [66] and ionic strength corrections were done as specified by Schindler et al. [37]. The results in Figure 7 should be taken as indicating the trends, because of the various uncertainties included from the parameter estimation, over the high ionic strength involved, to the use of predicted surface potentials in comparison to zeta-potentials.

## Competing interests

The authors declare that they have no competing interests.

## Authors' contributions

JL carried out the streaming potential measurements, performed the surface complexation modelling and wrote the draft manuscript. AA and JL carried out the SHG measurements. RW carried out the kaolinite measurements. RM did the thermodynamic calculations. FH performed the AFM measurements. VM provided the kaolinite sample, provided information on the kaolinite's properties and analyzed the specific surface area and the grain size. All authors were equally involved in discussing and interpreting the results, as well as in editing and commenting on the draft manuscript.
